# Emerging Functional Connections Between Metabolism and Epigenetic Remodeling in Neural Differentiation

**DOI:** 10.1007/s12035-024-04006-w

**Published:** 2024-02-10

**Authors:** Edgar Sánchez-Ramírez, Thi Phuong Lien Ung, Chiara Stringari, Lorena Aguilar-Arnal

**Affiliations:** 1 Departamento de Biología Celular y Fisiología, Instituto de Investigaciones Biomédicas, Universidad Nacional Autónoma de México, Mexico City, Mexico; 2 Laboratory for Optics and Biosciences, Ecole Polytechnique, CNRS, INSERM, Institut Polytechnique de Paris, Palaiseau, France

**Keywords:** Neural stem cells, Neurogenesis, Energy metabolism, Chromatin, Epigenetics, Transcriptional regulation

## Abstract

Stem cells possess extraordinary capacities for self-renewal and differentiation, making them highly valuable in regenerative medicine. Among these, neural stem cells (NSCs) play a fundamental role in neural development and repair processes. NSC characteristics and fate are intricately regulated by the microenvironment and intracellular signaling. Interestingly, metabolism plays a pivotal role in orchestrating the epigenome dynamics during neural differentiation, facilitating the transition from undifferentiated NSC to specialized neuronal and glial cell types. This intricate interplay between metabolism and the epigenome is essential for precisely regulating gene expression patterns and ensuring proper neural development. This review highlights the mechanisms behind metabolic regulation of NSC fate and their connections with epigenetic regulation to shape transcriptional programs of stemness and neural differentiation. A comprehensive understanding of these molecular gears appears fundamental for translational applications in regenerative medicine and personalized therapies for neurological conditions.

## Introduction

Stem cells (SCs) are undifferentiated cells that possess two unique features: self-renewal, which refers to the ability to divide and proliferate, generating identical cells while maintaining the characteristics of stemness, and potentiality, which is the ability to differentiate into different types of functional cells from daughter cells that have lost their stemness during the differentiation process [1]. Stem cells can be classified according to their differentiation capacity into totipotent, pluripotent, multipotent, and unipotent stem cells [2]. Pluripotent stem cells have the capacity to differentiate into all three germ layers,multipotent stem cells differentiate into a range of cell types within a specific tissue; and unipotent stem cells have the potential to produce only one cell type. Additionally, totipotent stem cells can be obtained in the early stages of embryonic development when the zygote divides to form the morula. These cells can differentiate into any type of embryonic and extra-embryonic tissue, and even generate a complete organism [2]. As embryonic development progresses, the self-renewal and differentiation potential of stem cells changes [3]. For example, embryonic stem cells (ESCs) are isolated from the inner mass of the blastocyst, which forms between days 5–6 of human embryonic development. These cells have pluripotent characteristics and can generate the three embryonic germ layers (mesoderm, ectoderm, and endoderm) but not extra-embryonic tissues [4]. Yet, stem cells are not limited to the embryonic stage and can also be found in adult tissues such as bone marrow, brain, and skin, which are referred to as adult stem cells. Neural stem cells (NSCs) are an example of adult stem cells, which reside in neurogenic niches within the adult brain [5].

NSCs are derived from the ectoderm during gastrulation and are a type of multipotent stem cell that generates different lineages of neurons, astrocytes, and oligodendrocytes during embryonic development. The central nervous system is formed from NSC lining the neural tube [6]. Subsequently, NSCs are “inactivated” at the end of the nervous system development, giving rise to quiescent NSC (NSCq). Thus, NSCq will be in a relatively inactive state until specific signals trigger their activation to participate in various physiological or pathophysiological functions in the brain, for example, neuronal plasticity, or repair of brain lesions [7]. The behavior of neural stem cells (NSCs), including their equilibrium between quiescent and active states, proliferation, and differentiation, is governed by a multitude of stimuli present in the NSC microenvironment, both during embryonic development and in the adult organism. For instance, growth factors such as fibroblast growth factor 2 (FGF-2), epidermal growth factor (EGF), or bone morphogenetic protein (BMPs) [8, 9] have demonstrated pivotal roles in regulating NSC proliferation and differentiation in embryonic and adult brains. Furthermore, extracellular matrix (ECM) proteins like laminin and fibronectin play a crucial role by offering structural support and modulating cell signaling [10]. In addition to ECM proteins, neurotransmitters such as dopamine and serotonin, which are known to influence the proliferation and differentiation of neural stem cells in the adult brain, contribute to this intricate regulatory network [11, 12]. The sophisticated interplay among these diverse factors within the NSC microenvironment significantly impacts NSC behavior, ultimately shaping neural development and repair processes [13, 14].

During development, NSCs initially expand by symmetric self-renewing divisions, in which NSC divides to produce two identical daughter cells, both of which retain their stem cell characteristics. Later, NSCs can divide via asymmetric division, originating either another NSC through self-renewing or neural progenitor cells (NPC) [15]. This population of cells is defined by their ability to proliferate through symmetric self-renewal divisions in a controlled manner for a limited period before expressing a differentiated phenotype [6]. The number of neurons generated during neurogenesis is determined by the initial number of neural stem/progenitor cells (NS/PC), the duration of their proliferative period, and their lineage. NSCs can generate all cell types of the brain, whereas NPC has a more restricted potential [16].

Differentiation of stem cells into specific cell types involves the expression of lineage-specific genes, inhibition of factors inducing differentiation into other lineages, and expression of factors promoting differentiation into a specific lineage [17]. Differentiation is regulated by extracellular signals, such as cytokine signaling, and intracellular programs such as epigenetic regulation, which involves DNA methylation, chromatin and histone modifications, functional non-coding RNAs, dynamic chromatin long-range interactions, and distinct chromatin spatial conformations, among other mechanisms [18, 19]. NS/PC must integrate cell-intrinsic programs and environmental signals in time and space to promote correct development [20], assisted by coordinated and subsequent gene expression programs regulated by transcription factors and the dynamic epigenome. Moreover, stem cell metabolism also plays a role in determining cell fate. Recent studies of stem cell metabolism have revealed coordinated fluctuations in energy metabolism and oxidative stress during NS/PC maturation and differentiation [21–23]. For example, the differentiation of NSCs into neurons shows significant changes in cell morphology and metabolism, such as the glycolytic activity which progressively decreases [24]. Interestingly, NSCs remain quiescent in a hypoxic niche with low O_2_ tension and obtain their energy by anaerobic glycolysis [25]. This glycolytic metabolism is substantially less energy efficient, suggesting that anaerobic glycolysis is not merely an environmental adaptation but an intrinsic necessity for quiescent adult stem cells. However, the differentiation of NPC to neurons is accompanied by a metabolic reprogramming, where the main source of energy will switch to oxidative phosphorylation (OXPHOS) [21, 23]. Remarkably, the metabolic state of stem cells can influence their epigenetic regulation. During differentiation, transitions form a quiescent state to a more active state implicate that metabolic preferences change. This metabolic shift, in turn, affects the availability of metabolites and cofactors necessary for epigenetic modifications, including DNA methylation and histone modifications [26]. For example, the acetyl-CoA produced during glycolysis is a critical substrate for histone acetylation, a key epigenetic modification that can activate gene expression. Changes in glycolytic flux can alter the levels of acetyl-CoA and, in turn, influence the epigenetic landscape to coordinate specific gene expression patterns.

Here, we review how certain metabolites playing an important role in energy and intermediary metabolism fluctuate during neural differentiation, and coordinately promote enzymatic reactions leading to epigenetic modifications or the activation of transcription factors that regulate specific transcriptional programs necessary for proper neuronal differentiation. We structured this review in two major sections, the first one oriented to outline metabolic transitions during NSC differentiation, and a second section describing advances on how these fluctuations in certain metabolites directly regulate specific epigenetic mechanisms contributing to the dynamic transcriptional reprograming. Finally, we describe technological approaches which could contribute to further expanding our knowledge in this molecular interplay. By delving deeper into these mechanisms, we can gain a greater understanding of how to harness neural differentiation for cell-based therapies and potentially unlock new avenues for the treatment of debilitating conditions affecting the brain and nervous system.

## Metabolic Fluxes Define Neural Stem Cell Fate

**Fig. 1:**
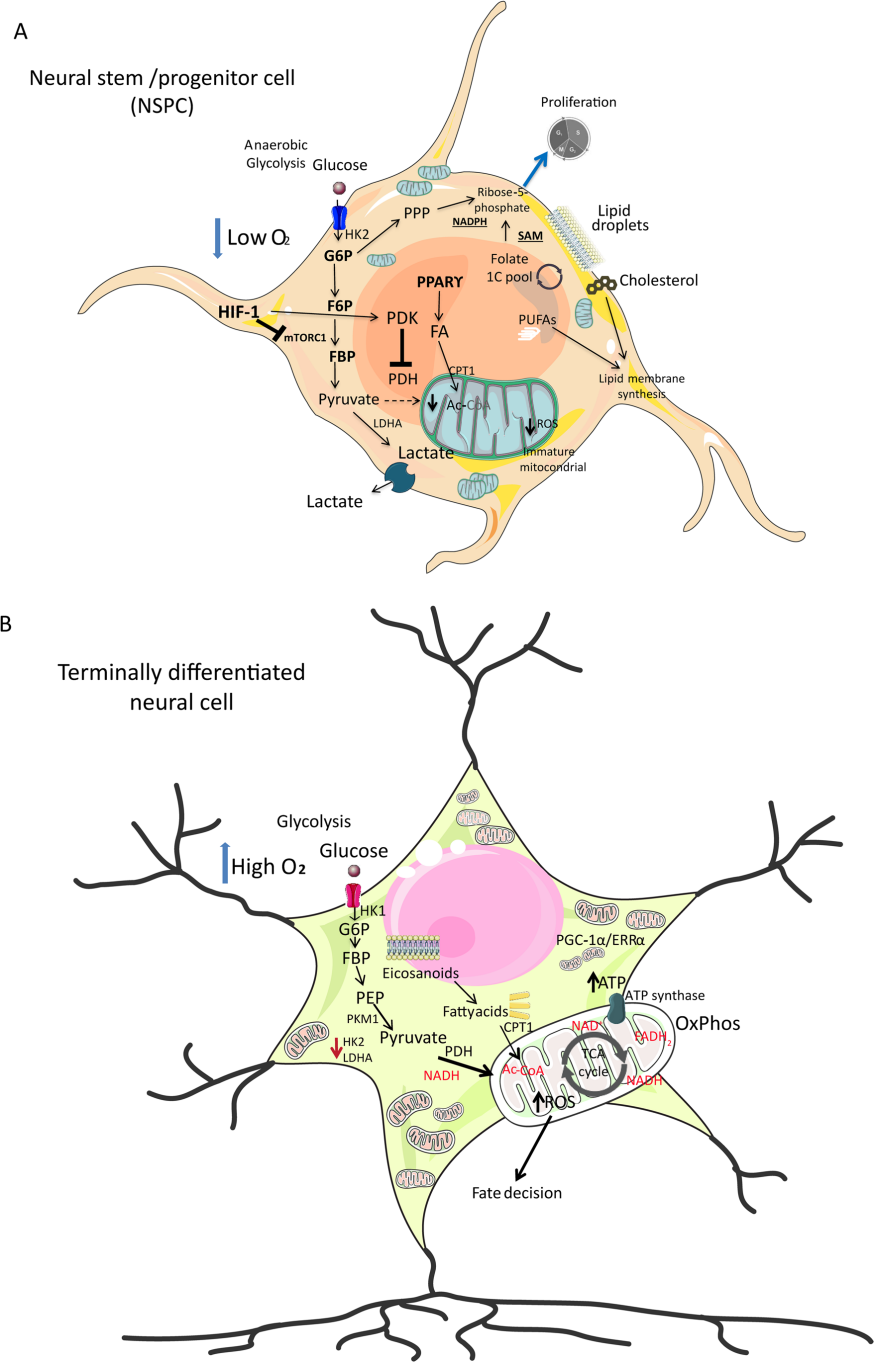
**A** Metabolism in neural stem and progenitor cells (NS/PC). In a hypoxic niche, neural stem cells (NSCs) maintain a quiescent state and predominantly rely on anaerobic glycolysis for energy production. These cells actively regulate reactive oxygen species (ROS) levels and exhibit immature or non-functional mitochondria. Neural progenitors (NPCs) utilize distinct metabolic pathways, including the pentose phosphate pathway and the folate 1C cycle, to support their proliferation. Furthermore, NPCs facilitate the synthesis of phospholipid membranes in collaboration with cholesterol biosynthesis, accompanied by elevated levels of lipid droplets essential for sustaining stem cell properties. **B** Metabolism of terminally differentiated neural cells. A notable metabolic reprogramming is evident, characterized by increased activity of glycolytic enzymes, such as hexokinase 1 and pyruvate kinase isoform 1, alongside reduced expression of hexokinase 2 and lactate dehydrogenase A. Simultaneously, pyruvate undergoes efficient conversion to acetyl-CoA for entry into the TCA cycle, where oxidative phosphorylation becomes the primary source of ATP. The upregulation of mitochondrial biogenesis, orchestrated by PGC-1α/ERRα, further accentuates this shift. Concurrently, lipid metabolism, encompassing eicosanoids and fatty acid oxidation, assumes a pivotal role in elevating acetyl-CoA levels, fueling the TCA cycle and augmenting NADH and FADH2 production critical for establishing the proton gradient driving ATP synthase. Concomitantly, elevated mitochondrial activity contributes to heightened ROS levels, playing a crucial role in determining cell fate. Abbreviations: glucose 6-phosphate (G6P); fructose 6-phosphate (F6P); fructose 1,6-bisphosphate (FBP); S-adenosilmetionina (SAM); acetyl-CoA (AcCoA); fatty acid (FA), reduced nicotinamide adenine dinucleotide phosphate (NADPH); polyunsaturated fatty acids (PUFAs); peroxisome proliferator-activated receptor gamma (PPARϒ); hypoxia-inducible factor 1(HIF-1); mammalian target of rapamycin complex 1 (mTORC1); pentose phosphate pathway (PPP); reactive oxygen species (ROS); phosphoenolpyruvic acid (PEP); nicotinamide adenine dinucleotide (NAD.^+^); reduced nicotinamide adenine dinucleotide (NADH); reduced flavin adenine dinucleotide (FADH2); fatty acid oxidation (FAO); oxidative phosphorylation (OxPhos); adenosine triphosphate (ATP); tricarboxylic acid cycle (TCA cycle); peroxisome proliferator-activated receptor gamma coactivator-1 alpha (PGC-1α); estrogen-related receptor alpha (ERRα). Enzymes: hexokinase 1 (HK1); pyruvate kinase isozymes M1 (PKM1); lactate dehydrogenase A (LDHA); pyruvate dehydrogenase (PDH); pyruvate dehydrogenase kinase (PDK), carnitine palmitoyltransferase 1 (CPT1); acetyl-CoA carboxylase (ACC). Parts of the figure were drawn by using pictures from Servier Medical Art by Servier, licensed under a Creative Commons Attribution 3.0 Unported License (https://creativecommons.org/licenses/by/3.0/)

Neuronal differentiation is a tightly controlled process through multiple regulatory layers. During the past few years, metabolic transitions are emerging as direct regulators of neural differentiation [27]. Accordingly, energy-regulating pathways are highly dynamic, showing flexibility to adapt in response to environmental factors including developmental signals [28]. Glycolysis in the cytoplasm and the tricarboxylic acid (TCA) cycle inside the mitochondria are coupled by pyruvate, through the pyruvate dehydrogenase complex, and their activity constitutes a major source of metabolic precursors and energy fluxes (Fig. 1) [29]. In fact, most terminally differentiated cells rely on the coordinated action between the TCA cycle and mitochondrial OXPHOS to meet energy demands. In this regard, dehydrogenases in the TCA cycle transfer electrons to the reducing equivalents nicotinamide adenine dinucleotide, NAD(H) and flavine adenine dinucleotide, FAD(H_2_), which in turn carry them into the electron transport chain to generate an electrochemical proton gradient across the inner mitochondrial membrane used by the ATP synthase to produce ATP [30]. Because the OXPHOS process consumes oxygen, in hypoxic conditions, mitochondrial function is inhibited and glucose is metabolized in the cytoplasm to lactate by lactate dehydrogenase (LDH), in a process known as anaerobic glycolysis. Surprisingly, many stem cell types rely on lactate production for their metabolic needs even in the presence of oxygen, a phenomenon known as the Warburg effect or aerobic glycolysis, first identified in cancer cells [31, 32]. Indeed, the incomplete oxidation of glucose to lactate permits a less efficient but faster rate of ATP production, but it also allows for the conservation of glycolytic intermediates which serve as substrates for a number of metabolic pathways whose fluxes are essential to meet the biosynthetic demands in highly proliferative cells, such as stem cells. For example, channeling glucose-6-phosphate, fructose-6-phosphate, and fructose-1,6-bisphosphate through the pentose phosphate pathway (PPP) generates NADPH and ribose-5-phosphate, which is necessary to sustain high rates of nucleic acid biosynthesis in proliferating cells [33]. Also, 3-phosphoglycerate can be shunted into the serine biosynthesis pathway, leading to the generation of serine, which is a precursor for proteins and participates in phospholipid biosynthesis. Definitively, it is clear that metabolic transitions occur during neural differentiation to support specific metabolic requirements at each stage.

### Metabolic Reprogramming in Neural Differentiation: From Glycolysis to OXPHOS

During neurogenesis, a switch from glycolytic to oxidative metabolic phenotype has been well characterized [27] (see Table 1). Evidence from a *Xenopus* model of retinal differentiation demonstrated that proliferating stages are highly dependent on glycolysis in vivo, with cells presenting higher levels of intracellular lactate and lactate dehydrogenase (LDH) and lower oxygen consumption rates than these cells at terminally differentiated stages (Fig. 1A, B) [34]. Importantly, a perturbation of this metabolic balance leads to impaired development of the retina. Similarly, in *Drosophila*, inhibiting OXPHOS in NSC impedes neurogenesis during development [35], van den [36]. Concomitantly, in human NPC, a transcriptional rewiring of metabolic genes accompanies and contributes to the metabolic switch during neurogenesis [23]. For example, downregulation of the glycolytic limiting enzymes hexokinase (HK2) and lactate dehydrogenase (LDHA) expression both at the mRNA and protein levels assists in transitioning from aerobic glycolysis in NPC to neuronal oxidative phosphorylation [23, 37, 38]. Alongside, hindering glycolysis through deletion of HK2 disrupts neural progenitor proliferation [37, 38].

**Table 1:**
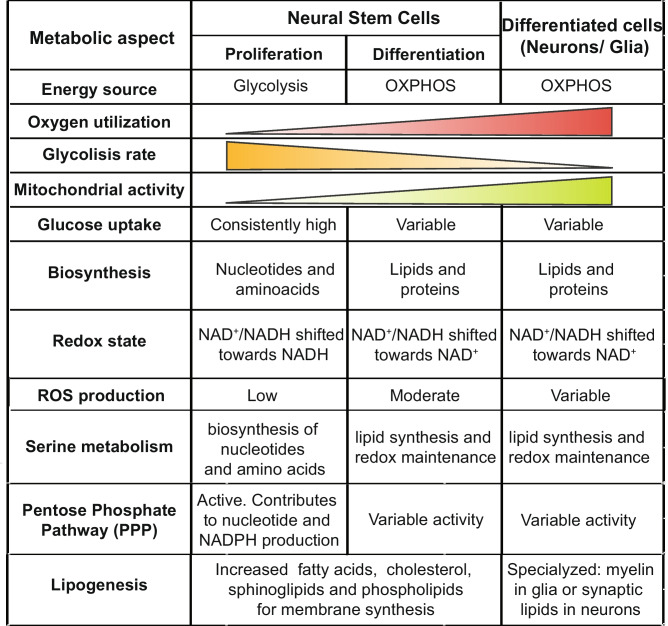
Metabolic transitions from neural stem cells to differentiated neural cells

As expected, increased OXPHOS during neurogenesis is tightly linked to mitochondrial function, and it has been postulated that mitochondrial dynamics regulate neural differentiation (Fig. 1). For example, during embryonic development in mice, NSC depicts elongated morphology, which becomes fragmented in committed neural progenitors, and regains an elongated shape in post-mitotic neurons. Disturbing these dynamics hinders stem cell self-renewal and neurogenesis, likely through dysfunctional metabolic fluxes [39, 40].

In human NSC, neural differentiation is accompanied by increased gene expression programs controlling mitochondrial biogenesis, including the peroxisome proliferator-activated receptor gamma coactivator 1-alpha (*PGC-1α*) and its downstream targets, *POLG*, *POLRMT*, *ERRα*, and *NRF1,* which is paralleled by decreased glycolytic flux (Fig. 1B) [41]. Accordingly, mitochondrial dysfunction as a result of a genetic or neurodegenerative disease drives cognitive decline, at least in part, through loss of adult NSC and neurogenesis, and leads to neuronal death [42–46]. Notably, enhancing mitochondrial function, for example overexpressing key proteins such as the inner membrane GTPAse OPA1, or the manganese superoxide dismutase SOD2, has been proven an effective strategy opposing neuronal decline in mice [47–50]. This evidence indicates that a tight balance between metabolic fluxes and mitochondrial function and dynamics controls NSC maintenance and neurogenesis.

Increased OXPHOS to provide ATP during differentiation consumes oxygen and generates high levels of reactive oxygen species (ROS). It is well established that ROS production has a profound impact on neural differentiation. For example, low ROS levels below 1 nM H_2_O_2_ contribute to maintaining a quiescent state in stem cells, while increasing ROS promotes self-renewal and differentiation [51]. However, above 10 nM H_2_O_2_, oxidative stress increases leading to cell death and neural tissue degeneration. Physiological fluctuations in ROS levels contribute to neurogenesis, maturation, and synaptic plasticity, although the mechanisms are not fully understood [52, 53]. Notably, mitochondria serve as the primary source of ROS, influencing mitochondrial dynamics and potentially leading to the formation of granular mitochondria [54]. Concurrently, changes in mitochondrial shape can impact mitochondrial function and redox status, amplifying ROS production and oxidative stress [55]. Mitochondrially derived ROS function in redox signaling, but in excess, they contribute to cellular injury and death. Importantly, the accumulation of mitochondrial ROS affects neural cell fate by inducing oxidative stress and DNA damage and activating cell death pathways, while alterations in mitochondrial dynamics influence cellular bioenergetics, differentiation, and fate determination [56, 57]. This evidence underscores the intertwined roles of mitochondrial dynamics and ROS in shaping neural cell fate, while the implicated mechanisms need further exploration.

It is generally assumed that stem cells reside in hypoxic niches which might contribute to sustaining glycolytic metabolism; however, evidence for low oxygen tension in adult NSC niches is scarce [58]. Notably, adult NSCs in the brain, such as those in the subventricular zone (SVZ) and the dentate gyrus of the hippocampus, exist in a more oxygenated environment compared to embryonic NSCs. Local oxygen levels in the neurogenic niches where adult NSCs reside can influence their behavior. For example, normoxia can stimulate the activation of adult NSCs and their differentiation into neurons or glial cells. The glycolytic phenotype of NSC and NPC, characterized by a preference for glycolysis over oxidative phosphorylation to promote energy-efficient maintenance of their quiescent state (NSC) or for energy production (NPC), renders them highly resistant to hypoxic conditions [59]. Interestingly, numerous data support that low oxygen tension increases self-renewal and proliferation of NSC in vitro, and defines cell fate [60–65]. Several molecular mechanisms are implicated. For example, progressive relief of hypoxia during the development of the mouse cerebral cortex shapes NSC differentiation in vivo, a mechanism involving a gradual decline in hypoxia-inducible factor (HIF)-1α signaling [66]. Importantly, the transcription factor HIF-1α is stabilized under hypoxic conditions, at oxygen levels below approximately 6 to 2% oxygen (or 60–20 mm Hg) [67], and controls a transcriptional program of genes which ultimately promote glycolysis and repress OXPHOS [68]. HIF-1α is essential for NSC maintenance and contributes to adult neurogenesis [69, 70], and also influences important pathways such as Wnt/β-catenin and Notch function, which fine tune NSC proliferation, differentiation, and neuronal maturation (Fig. 1A) [71–74]. HIF-1 also promotes overexpression of its downstream target *BNIP3*, which in turn inhibits mTORC1 activity, hereby increasing autophagy which appears necessary for NSC proliferation [75]. Another advantage of hypoxia-induced glycolysis in NSC is the decreased ROS production as compared to terminally differentiated neurons relying on OXPHOS. In fact, raising ROS during neural differentiation constitutes a signaling event influencing NSC fate decision. For example, ROS increase the activity of the transcription factor NRF2, a master regulator of the antioxidant response, which also targets the expression of pro-neuronal factors such as Isl1, Nkx2.1, Lhx5, and Sim1 [40]. Along these lines, maintaining low ROS levels in NPC is also influenced by the forkhead box O (FoxO) family of transcription factors, which tightly control metabolic fluxes and sustain redox balance in these cells [76, 77], and autophagy rates in developing and adult neurons [78]. Recent research reveals a notable divergence in energy metabolism among neurons based on subcellular structures. Specifically, somata exhibit elevated aerobic glycolysis and reduced OXPHOS compared to terminals [79]. Further investigations are needed to clarify the physiological significance of this metabolic compartmentalization and the underlying mechanisms.

In summary, cumulative evidence suggests that a shift in metabolic pathways from glycolysis to OXPHOS takes place during neural differentiation. The relationship between oxygen levels and metabolic shifts during NSC differentiation and proliferation is bidirectional: low oxygen (hypoxia) promotes glycolysis and proliferation by activating HIF-1α to maintain stemness and support rapid cell division, while higher oxygen levels (normoxia) are associated with the transition to OXPHOS, which supports differentiation, and the increased energy demands of terminally differentiated neural cells. These transitions are meticulously regulated and synchronize with gene expression programs and intracellular signaling. Collectively, these mechanisms sustain the self-renewal and proliferative potential of NSC and play a role in determining cell fate and shaping neurogenesis and homeostasis in adult neurons.

### Fatty Acid Metabolism in Neural Stem Cells

Besides glucose fluxes, fatty acid metabolism plays a crucial role in the biology of NSC, regulating proliferation, differentiation, and self-renewal. The eicosanoid pathway and fatty acid synthesis, as well as fatty acid oxidation (FAO), contribute to neurogenesis at distinct stages [80]. In neural cells, phospholipids such as phosphatidylcholine, phosphatidylethanolamine, and phosphatidylserine are essential for membrane structure and function [81]. Glial cells require sphingomyelin and glycosphingolipids, particularly for constituting myelin sheaths. Gangliosides, a type of glycosphingolipid with sialic acid residues, are essential for neuronal development, signaling, and synaptic function [82]. Additionally, lipid droplets, the lipid-storing organelles, are highly abundant in mouse NPC and decay during differentiation [83]. Increased lipid droplets promote proliferative and differentiation capacities and fuel FAO to sustain NPC survival [83]. It is generally accepted that the brain mostly relies on glucose as a source of energy over lipids and FAO, hereby avoiding ROS production and oxidative damage [84]. However, FAO is now emerging as a critical regulator of neural development [85]. For example, quiescent mouse NPC expresses high levels of the FAO rate-limiting enzyme carnitine palmitoyltransferase I (CPT1), and FAO is more active than in proliferative NPC and mature neurons. Inhibiting CPT1 leads to defective neurogenesis and cell death, while stimulating it alters NPC proliferation (Fig. 1) [86, 87]. Additionally, FAO is a critical regulator of the transition from mouse NSC to intermediate progenitor cells during brain development, which may underlie defective cell division leading to autism spectrum disorder (ASD) [88]. In fact, fatty acids serve as energy sources, but can also be incorporated into various lipid molecules such as long-chain polyunsaturated fatty acids like docosahexaenoic acid (DHA), which is particularly important for neural development and function. DHA is an omega-3 fatty acid and is the major prevalent fatty acid in the brain membrane [89]. The brain maintains its fatty acid levels mainly via the uptake of plasma-free DHA, and its metabolization to N-docosahexaenoylethanolamide leads to neurite growth, synaptogenesis, and synaptic function, which is relevant for learning and memory [90].

Aberrations in lipid metabolism can have significant consequences for neural development, homeostasis, and function, contributing to various neurological disorders. For example, abnormal oleic acid-enriched triglyceride accumulation within ependymal cells, the main support cells of the forebrain NSC niche, during brain development might promote Alzheimer’s disease (AD) in mice [91], underscoring the relevance of lipid metabolism in neural development.

On the other hand, de novo lipogenesis is catalyzed by the fatty acid synthase (FASN) enzyme, which is elevated in mouse NSC when compared to mature neurons [92]. FASN activity is required for normal NPC proliferation and neurogenesis [92–94]. In mice and humans, a *FASN* R1819W mutation impairs adult hippocampal NSPC activity and cognitive defects due to lipid accumulation and lipogenic ER stress in these cells [93, 94], reinforcing the idea that lipid metabolism contributes to cognitive function [95].

Certain lipids appear to be involved in neurogenesis (Fig. 1). For example, oleic acid is an endogenous ligand for the nuclear receptor NR2E1/TLX, which controls NSPC self-renewal and proliferation. When bound to oleic acid, TLX becomes a transcriptional activator of neurogenic genes, driving hippocampal development in mice [96]. Along the same lines, omega-3 polyunsaturated fatty acids (n-3 PUFAs) are emerging as neuroprotective compounds [97], and recent research shows that in a human hippocampal progenitor cell line, they exert a transcriptional response for pathways involved in oxidative stress and inflammation [98]. Importantly, the nuclear receptor peroxisome proliferator-activated receptor alpha (PPARα) in the brain binds a class of omega-3 hydroxy fatty acid promoting multiple dendritic responses in mouse cortical neurons, which might result in neuroprotective effects [99].

Lastly, cholesterol metabolism plays a central role in the central nervous system. It is considered that the brain contains about 25% of the whole body’s cholesterol [100]. It contributes to maintaining membrane fluidity and modulating the function of membrane proteins and, importantly, is involved in myelin formation [101]. Because cholesterol is a key component of membranes in mammalian cells, neural functions are tightly linked to cholesterol bioavailability, including the formation of synapses [102]. Several diseases are associated with defects in cholesterol metabolic pathways, such as Alzheimer’s disease, Huntington’s disease, and Parkinson’s disease [103]. Interestingly, selective ablation of cholesterol biosynthesis in NSC in the adult brain is accompanied by increased VEGF expression, which promotes angiogenesis to supply the niche with circulating cholesterol-loaded lipoproteins [104]. Yet, in the developing brain, altering cholesterol biosynthesis leads to accelerated neurogenesis and abnormal migration and proliferation patterns of cerebellar granule precursors [105, 106].

### Folate-Dependent One-Carbon Metabolism Is Critical to the Developing Brain

Mounting research highlights an essential role for folate-dependent one-carbon (1C) metabolism in the developing brain [107, 108]. 1C metabolism consists of the folate cycle, the methionine remethylation, and the transsulfuration pathways, which are essential for amino acid metabolism, purines and thymidine biosynthesis, and synthesis of S-adenosylmethionine (SAM), the universal methyl donor for methylation reactions [107–109]. A strong association between folate-1C metabolism deficiencies and defects in neural tube development has been established, which generally leads to adverse pregnancy outcomes [110]. In this regard, many GWAS studies have identified certain SNPs in genes encoding rate-limiting enzymes in 1C metabolism, such as the methylenetetrahydrofolate reductase (*MTHFR*) or betaine-homocysteine S-methyltransferase 1 (*BHMT1*), associated with increased risk of neural tube defects during development [111], Rampersaud, [112, 113]. Mounting research has emphasized the significance of folate in the self-renewal and differentiation of neural stem cells (NSCs). Inadequate levels of folate have been found to impede the growth of NSCs in the adult hippocampus in vivo and prompt NSC cell death in vitro [114, 115]. Accordingly, NSCs exhibit increased proliferation and neuronal differentiation in response to folate [116, 117].

## Epigenetic Regulation and Metabolic Fluxes Intersect to Shape Neurogenesis

As related, neural fate commitment and differentiation are typically accompanied by significant transitions in metabolic activity, as specific pathways assume control over the supply of fuel. These metabolic transitions are accompanied by extensive transcriptional rewiring of gene regulatory networks to sustain the differentiation process [118, 119]. Indeed, transcription occurs in the chromatin fiber,hereby, chromatin states are key controlling neural development. As a result, distinct modifications on chromatin conform to an epigenetic regulatory landscape shaping transcriptional transitions in neural development [120].

During neural commitment, ESCs lose their pluripotency through activating epigenetic mechanisms leading to chromatin transitions in regulatory regions of multiple genes. Extrinsic signaling promotes the expression of proneural genes such as *Sox1*, *Pax6*, and *Ngn1* through chromatin remodeling at their regulatory elements, while genes activated only in terminally differentiated cells remain poised until their cell fate is defined [121, 122]. While technological advances in high-throughput sequencing have contributed to defining these transcriptional and chromatin transitions with high precision and even to a single-cell level, the interplay between metabolic and epigenetic reprogramming and how it evolves with time during neurogenesis is emerging.

Metabolism and epigenetics intersect at the level of metabolites that are used as substrates and cofactors for enzymes catalyzing reactions to directly modify chromatin and hereby regulate gene expression [123]. Certain metabolites such as acetyl coenzyme A, SAM, NAD^+^, FAD, or even glucose and lactate are necessary for the activity of chromatin modifiers that define the epigenetic landscape [124]. Since the availability of these metabolites relies on the intermediary metabolism, the wide metabolic fluctuations observed during neural differentiation are thought to impact the epigenetic landscape.

In the next sub-sections, we describe current knowledge of this direct interplay between metabolic and epigenetic transitions to control gene expression in neurogenesis. Of note, a comprehensive view of epigenetic and epigenomic reprogramming in neurogenesis is not the focus of this review and has been extensively reviewed elsewhere [125, 126], which we recommend to the reader aiming to get a complete understanding on this specific topic.

### SAM-Directed DNA Methylation Contributes to Neural Development

DNA methylation is a pivotal process that plays a crucial role in development through controlling gene expression, genomic imprinting, or chromatin structure. It involves the transfer of the methyl group from the metabolite SAM to a cytosine by DNA methyltransferase enzymes (DNMTs), forming 5-methylcytosine (5mC). Moreover, there is a distinct form of DNA methylation known as 5-hydroxymethylcytosine (5hmC) that occurs frequently in the mouse brain. During embryonic development, de novo DNMTs DNMT3A and DNMT3B establish the DNA methylation pattern, while DNMT1 maintains this pattern. DNA methylation can be removed passively by blocking methylation of newly synthesized DNA during replication or through enzymatic reactions that remove the methyl-modifications [127] such as the ten-eleven translocation (TET) family [128]. DNA methylation regulates gene expression via preventing transcription factor binding and silencing genes through recognition by transcriptional repressors with a methyl-CpG-binding domain (MBD) such as MBD1 and methyl-CpG binding protein 2 (MeCP2)[129]. Importantly, extensive methylome reconfiguration occurs during mammalian brain development, which is cell-type specific and modulates the function of regulatory regions in the genome [130]. For example, during embryonic neural development, the DNA methylation status of genes such as *Pax6*, which codes for a transcription factor, can determine cell fate [131]. Methylation of the *Pax6* gene regulatory elements regulates its activity and promotes neurogenesis from ESC [132]. Recent literature supports the idea that metabolites such as folate and SAM can regulate DNA methylation in neural development. For example, during axonal myelination in mice, global DNA demethylation occurs, which is associated with the activation of genes such as *Srebf1*, *Hmgcr*, *Dgat1*, *Alc27a1*, and *Abca2*, which are crucial to activate lipid metabolism required for myelination process [133]. Interestingly, SAM levels appear reduced during Schwann cell myelination, and increased SAM leads to abnormal hypermethylation and peripheral nerve defects [133].

**Fig. 2:**
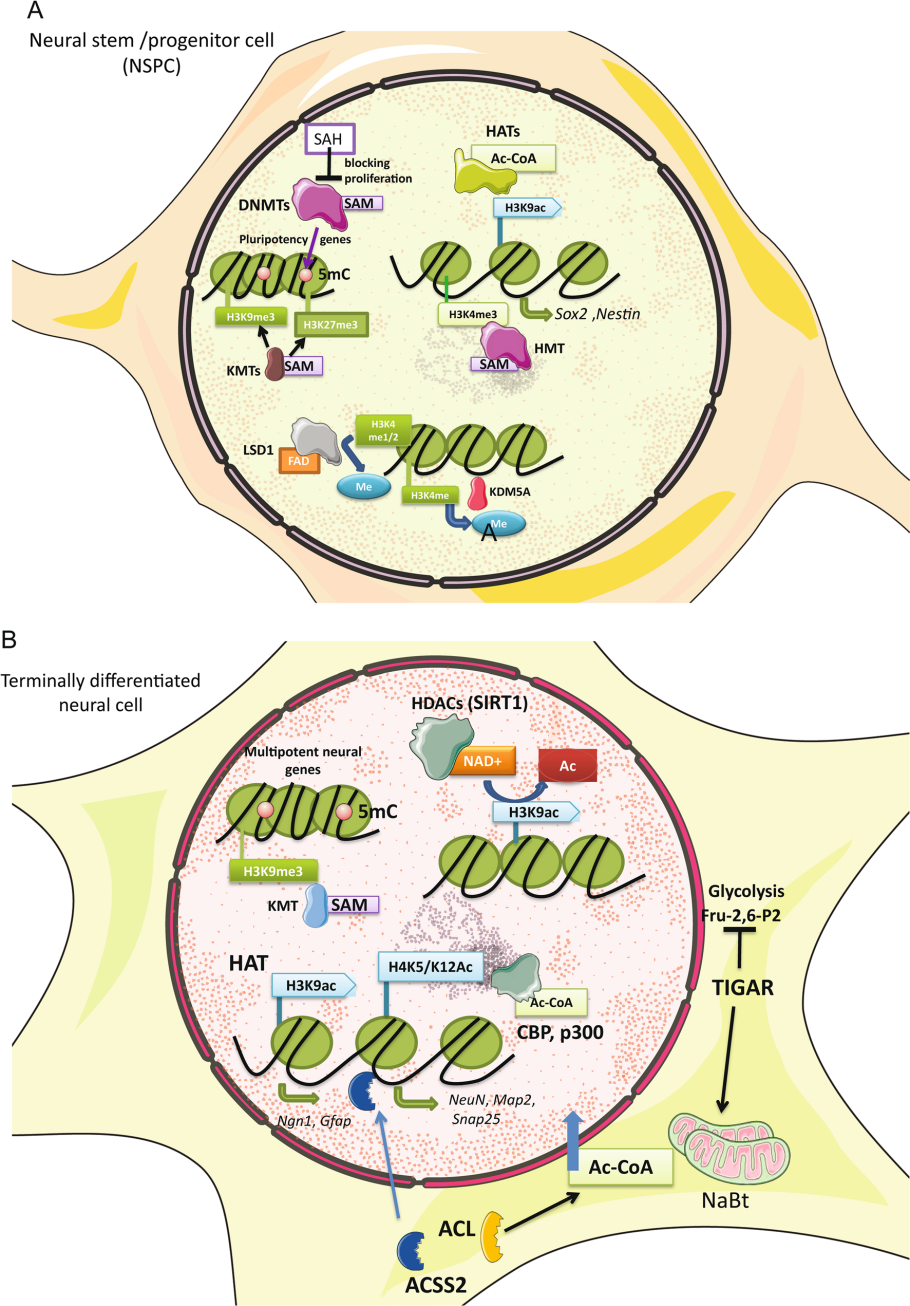
The interplay between metabolism and the epigenome in neural stem/progenitor cells and terminally differentiated neural cells. **A** Metabolism plays a crucial role in establishing and maintaining the epigenetic state that underlies the neural stem/progenitor cell phenotype. Enzyme-mediated post-translational modifications of DNA or histones relying on small metabolites as cofactors contribute to this process. Repressive DNA marks, such as 5mC, catalyzed by DNMTs, and histone methylation marks H3K9me3 and H3K27me3, catalyzed by SAM-dependent KMTs, silence pluripotency genes. Conversely, the presence of H3K9ac, catalyzed by acetyl-CoA-dependent HATs, activates certain genes. The maintenance of the multipotent state is favored by FAD-dependent LSD1 and the KDM5A protein. **B** In terminally differentiated cells, metabolism, particularly oxidative metabolism, promotes an acetyl-CoA pool, which facilitates the activity of HAT, CBP, and p300, leading to an active transcriptional state for differentiation genes. TIGAR inhibits glycolysis, whereas NaBt promotes mitochondrial biogenesis. Multipotent genes are transcriptionally repressed by DNA and histone repressive marks. In addition, the NAD^+^-dependent deacetylase SIRT1 targets H3K9ac, promoting transcriptional repression at specific genes which determine cell fate. Abbreviations: NAD.^+^, nicotinamide adenine dinucleotide; acetyl-CoA, acetyl coenzyme A; SAM, S-adenosylmethionine; SAH, S-adenosyl-L-homocysteine; FAD, flavin adenine dinucleotide; HAT, histone acetyltransferases; DNMTs, DNA methyltransferases; KMT, lysine methyltransferase; LSD1, lysine-specific demethylase 1; 5mC, 5-methylcytosine; Me, methyl group; SIRT1, NAD-dependent deacetylase sirtuin-1; HDAC, histone deacetylases; ACSS2, acetyl-CoA synthetase 2; ACL, ATP-citrate synthase; TIGAR, TP53-inducible glycolysis and apoptosis regulator; NaBt, sodium butyrate; CREB binding protein (CBP); p300, histone acetyltransferase p300; Ac, acetyl group. Parts of the figure were drawn by using pictures from Servier Medical Art by Servier, licensed under a Creative Commons Attribution 3.0 Unported License (https://creativecommons.org/licenses/by/3.0/)

The relationship between folic acid metabolism and neural development has been largely known; however, the direct impact of 1C metabolism on the DNA methylation dynamics in neurogenesis is still a matter of debate [134, 135]. In humans, evidence of a direct impact of 1C metabolism intermediaries in controlling DNA methylation during neurogenesis is scarce. A remarkable study was conducted on the *POMC* gene which encodes several peptides involved in the appetite control in the hypothalamus [136]. *POMC* methylation status in the brain from post-mortem subjects was positively correlated with individual weight. Interestingly, lower *POMC* methylation was found in children conceived from mothers with a lower supply of 1C metabolites, while a robust negative correlation for SAH and positive correlations with betaine and SAM were found at several *POMC* promoter methylation sites [137]. Notably, the availability and dynamics of 1C metabolism intermediaries have been implicated in the development of a number of neurodegenerative conditions such as Alzheimer’s disease, Huntington’s disease, and autistic spectrum disorders [138–140]. In rodents, mounting evidence sustains a direct relationship,for example, in NSCs, folate treatment leads to increased proliferation while inhibition of DNMT1 counteracts it [141, 142]. This is paralleled by differential methylation of a set of genes involved in critical pathways such as neuroactive ligand–receptor interaction, Jak-STAT signaling pathway, steroid biosynthesis, fatty acid elongation, PI3K/Akt, MAPK signaling pathway, and cytokine–cytokine receptor interaction [141]. Furthermore, a low folate diet alters the 5mC and 5hmC epigenetic patterns in mESC, altering the expression of neural development genes pertaining to the hedgehog signaling pathway which is involved in the formation of the neural tube [143]. Concomitantly, severe maternal undernutrition leads to reduced proliferation and differentiation in NSC, with decreased levels of DNMT1 and altered expression of neural markers such as *Nestin* or *Hes1* [144]. Along the same lines, increased levels of S-adenosylhomocysteine (SAH), a bypass metabolite from SAM consumption during methylation reactions, inhibit methyltransferases, blocking proliferation in NPCs (Fig. 2A) [145]. Increased SAH leads to hypomethylation at the *Sprouty2* (*Spry2*) promoter and its overexpression, which negatively regulates the fibroblast growth factor receptor (FGFR)-Erk1/2-cyclin E signaling pathway and, hereby, impairs proliferative signaling [146].

### Metabolic Control of Histone Methylation During Neural Differentiation

Similar to DNA, methylation of histones consists of the transfer of a methyl group from SAM to a lysine or arginine residue of a histone protein, by histone methyltransferase (HMT) enzymes. Pioneering studies within the past decade demonstrated that SAM accumulation in mouse embryonic stem cells (mESC) selectively influences trimethylation of histone H3 lysine 4 (H3K4me3) levels, a transcriptionally activating mark involved in the maintenance of pluripotency [147]. Similarly, methionine-deprived human pluripotent stem cells (hPSC) also show decreased SAM and H3K4me3, which is accompanied by impaired self-renewal [148]. Interestingly, when induced to neural differentiation, methionine-deprived hPSC express higher levels of the neural marker *MAP2*, suggesting increased differentiation potency [148]. This evidence supports the idea that the metabolome regulates the epigenetic landscape in multipotent cells and during differentiation. In addition, at very early stages in human development, the repressive H3K27me3 and H3K9me3 marks are responsive to variations in SAM levels (Fig. 2B) [149].

Interesting research has recently demonstrated a link between one-carbon metabolism and histone methylation which determines neural differentiation. Using NSC, the authors demonstrated that activation of the ephrin receptor Eph-B induced downregulation of the transcript *Dhfr*, encoding a key enzyme in the 1C metabolic pathway [150]. Decreased DHFR expression and activity depletes the progenitor pool and promotes differentiation through an epigenetic switch consisting of decreased H3K4me3 levels on progenitor-specific genes such as *Sox2* and *Nestin* [150]. Together, these data indicate that the modulation of metabolic pathways influences stem cell fate through epigenetic modulation.

On the other hand, histone demethylation can be catalyzed by the JMJC (Jumonji-C) family of lysine demethylases (KDM). Among them, KDM5A, KDM6A, and KDM3A have been described as effective oxygen sensors [151–153]. KDM5A contributes to the maintenance of NPC by suppressing genes related to astrocyte differentiation [154]. KDM6A participates in resolving bivalent promoters, a particular class of chromatin enriched in both H3K4me3 and H3K27me3, during human neural differentiation [155], and activates specific neurogenic genes [156]. Also, KDM3A facilitates recruitment to chromatin of the transcription factor Neurog2, which is essential for neurodevelopment [157]. Hereby, it is tempting to speculate that the hypoxia signaling might coordinate dynamic changes in the epigenome shaping the neurogenic program. Further research is necessary to clarify this possibility.

Another kind of histone demethylases is the flavin adenine dinucleotide (FAD)-dependent lysine-specific histone demethylase (LSD) proteins. LSD1 removes mono- or di-methylation on lysine 4 of histone H3 (H3K4me1/me2) and also demethylates H3K9 or H4K20 [158–160]. In rodents, LSD1 supports the maintenance of NS/PC identity and proliferation, and a progressive decrease in LSD1 levels is required for neural differentiation [158, 161]. Interestingly, the pharmacological increase of FAD activates LSD1 in NSC, which is sufficient to induce neuronal differentiation [162]. Hereby, it is possible that histone demethylation, regulated by FAD-dependent LSD1 activation, may contribute to the regulation of neural differentiation coordinated by metabolic transitions (Fig. 2A).

### Histone Acetylation Is Responsive to Metabolic Cues

Modulation of histone acetylation is a fundamental process to define the fate of neural progenitors toward a specific lineage during neural development [163, 164]. It consists of the covalent addition of an acetyl group from acetyl coenzyme A (AcCoA) to a lysine residue on a histone by a histone acetyltransferase (HAT) enzyme, hereby neutralizing the positive charge to weaken DNA-histone interactions. Histone acetylation can be reverted by histone deacetylases (HDACs), leading to transcriptional repression. Mounting research illustrates the critical role of histone acetylation in the control of the neurogenic transcriptional programs and neurodegenerative conditions [165–167]. Interestingly, this epigenetic mark is widely responsive to metabolic cues [168], and the implications of this crosstalk for neurogenesis are beginning to emerge.

#### Histone Acetylation in Neurogenesis Depends on AcCoA

In mammals, two main enzymes sustain AcCoA production for histone acetylation: acetate-dependent acetyl-CoA synthetase 2 (ACSS2) and citrate-dependent ATP-citrate lyase (ACL). Unexpectedly, during neural differentiation, ACSS2 locates to the nucleus and regulates the expression of neural markers such as *NeuN*, *Map2*, or *Snap25* [169]. ACSS2 is recruited to chromatin sites proximal to genes linked to neural differentiation, which are also enriched for acetylation of lysines 5 and 12 of histone H4 (H4K5/K12Ac) and lysine 9 of histone H3 (H3K9Ac). This is consistent with the idea that ACSS2 locally provides a constant AcCoA fuel for histone acetylation by HATs CBP and p300 during neurodevelopment at neuron-specific genes (Fig. 2). Consistently, altering ACSS2 function in the hippocampus impairs long-term memory consolidation in mice [169]. Interestingly, oxygen and serum limitation increases nuclear localization of ACSS2 which contributes to sustaining histone acetylation levels and cell survival [170, 171]. Along these lines, recent research in mice demonstrates that alcohol intake is metabolized in the liver to acetate, which impacts the brain metabolism leading to increased histone acetylation and transcriptional activity in the dorsal hippocampus in an ACSS2-dependent manner [172]. This evidence illustrates important connections between metabolism and histone acetylation in neural development, with potential implications for cognitive conditions such as addictive behavior or age-associated memory decline.

We have previously discussed that during neural development, a switch from glycolytic to oxidative phenotype occurs. This metabolic rewiring might be connected to the wide epigenomic transitions driving neurogenesis. For example, TIGAR is a bisphosphatase which reduces intracellular fructose-2,6-bisphosphate levels, leading to decreased glycolytic flux [173]. Accordingly, TIGAR expression increases during embryonic cortex development and along the course of differentiation of NSC, which is accompanied by increased expression of neural markers such as MAP2, GFAP, *Tuj1*, *NeuroD1*, and *Ngn1* [174]. Interestingly, overexpression of TIGAR in NSC promotes differentiation favoring the glycolytic-to-oxidative metabolic switch, which is accompanied by increased AcCoA, which ultimately contributes to increased H3K9Ac at the promoters of critical neural genes including *Ngn1*, *Neurod1*, and *Gfap* (Fig. 2B*)* [174].

#### Histone Deacetylation in Neurogenesis and Its Interaction with Metabolism

HDAC activity manipulation has been linked to the metabolic reprogramming driving neural differentiation. For example, pan-HDAC inhibitors such as sodium butyrate (NaBt) or valproic acid (VPA) induce neuronal differentiation of NPC [175, 176]. NaBt promotes mitochondrial biogenesis and enhances the oxidative metabolism in NPCs [164]. This is accompanied by increased CBP HAT activity and relocation of the H3K27Ac mark at regulatory regions of genes involved in mitochondrial metabolism and OXPHOS, providing a mechanistic link between epigenetic and metabolic reprogramming in neurogenesis [164]. Similarly, VPA suppresses the proliferation of NPCs and promotes the expression of neural markers including Tuj1 and CaMKII. VPA also increases the glycolytic flux in NPCs and the H3K9Ac mark at the promoters of *Ngn2* and *Mash1*, and H4Ac within the *Ngn1*, *Math1*, and *p15* promoters [56, 57, 176].

Another deacetylase that is influenced by metabolism is the class III HDAC SIRT1. The deacetylase activity of SIRT1 is coupled to the hydrolysis of the metabolite NAD^+^, and has been characterized as a redox sensor, as SIRT1 activation is dependent on the NAD^+^/NADH ratio. The glycolytic phenotype of NSC tends to reduce the NAD^+^ pool, a process that intensifies with aging [177]. Interestingly, the pharmacological increase of NAD^+^ in aged mice promotes proliferation and neurogenesis in the hippocampus [177, 178], partially mediated by SIRT1 [177, 179]. Furthermore, the redox state is a critical regulator of self-renewal and differentiation of NPCs through controlling SIRT1, which when activated downregulates the H3K9Ac epigenetic mark, hereby dictating cell fate decisions (Fig. 2B) [180, 181]. Accordingly, mitochondrial impairment through increased oxidative stress and accumulation of mtDNA damage in NSC activates SIRT1 to epigenetically support *Mash1*-mediated astrogliosis [182], or ultimately drive cellular senescence and mitophagy [94]. Together, these findings indicate that SIRT1 is an important regulator of NS/PC self-renewal and differentiation potential, and an epigenetic effector of metabolic transitions in redox states [183].

## Novel Methods to Study Metabolic Transitions in Live Cells: Fluorescence Lifetime Imaging Microscopy of Endogenous Biomarkers

We have shown that small molecule metabolites play much underappreciated roles in cell differentiation and homeostasis by controlling the activity of several epigenetic remodelers. Generally, metabolic states are determined from whole cell lysates. Yet, local concentrations of certain metabolites could alter enzymatic properties in discrete microenvironments as shown in the case of ACSS2 locally supplying AcCoA. A redox switch from glycolytic to oxidative metabolism and wide epigenetic reprogramming are hallmarks for NSC maintenance and cell fate. However, the molecular links between these major processes during differentiation remain largely unknown. Hereby, novel approaches to measure metabolism across scales are very much needed in this field. To approach this challenge, it is necessary to consider cellular heterogeneity, which is an intrinsic mechanism of determination of cell fate. Deciphering heterogeneity has proven crucial to understanding differentiation processes, and single-cell transcriptomics has become the gold standard to untangle heterogeneity at the transcriptional level. While mounting research is seeding light into transcriptional and epigenetic regulation of neurogenesis in single cells, disentangling metabolism at the cellular level has fallen behind due to the limited availability of technological approaches. Currently, single-cell metabolomics is a rapidly advancing field. The main techniques used for single-cell analysis include mass spectrometry (MS) and MS imaging (MSI), electrochemistry, fluorescence microscopy, vibrational spectroscopy, and capillary electrophoresis, which have been thoroughly reviewed elsewhere [184–187].

A number of these techniques have been applied to reveal the metabolic intricacies of neural development. For example, a single-cell profiling method named matrix-enhanced-secondary ion mass spectrometry (ME-SIMS) was used to investigate the lipid profiles of neuronal cells, allowing to classify single-cell populations and subpopulations using SIMS profiling of lipid and metabolite contents [188]. Additionally, MSI has been proven effective in characterizing metabolism in human brain organoids, allowing direct comparison of certain lipid signal intensities and distributions, including ceramides and phosphatidylethanolamine [189].

In the last decade, optical metabolic imaging has been extensively applied to quantify redox states at subcellular scales. Two-photon excited fluorescence (2PEF) microscopy-based techniques provide functional images of tissues and cells through intrinsic fluorophores that are naturally present in cells and tissues [190]. Importantly, the metabolic coenzymes NADH and FAD constitute biomarkers for metabolism, mitochondrial function, and oxidative stress in live cells and tissues, due to their intrinsic fluorescent properties [191, 192]. Since the pioneering work of Britton Chance [193], NADH and FAD fluorescence intensity has been extensively used to monitor changes in metabolism in different fields of biomedical research such as neuroscience and differentiation [194, 195].

Multiphoton microscopy, such as third harmonic generation (THG), has been successfully used to label-free visualization of structures with high lipid content, including myelin sheaths, cellular membranes, or ECM structures in live organisms [196–198]. Two-photon fluorescence lifetime microscopy (2P-FLIM) of the metabolic coenzymes NADH and FAD has been increasingly used in recent years to perform metabolic imaging as it reveals the richness and complexity of several metabolic processes in a label-free and non-invasive way. For example, 2P-FLIM of NADH provides very sensitive measurements of the redox states (NADH/NAD^+^) of cells as well as glycolysis, OXPHOS, and oxidative stress rates [199–204] and has the spatiotemporal resolution required to characterize dynamic physiological states and NADH intracellular subcellular compartmentalization [183, 205, 206]. In recent years, fluorescence lifetime imaging of NADH has been increasingly used to monitor metabolic shifts in neurodegenerative diseases [207] as well as during stem cell differentiation, for example, to map subcellular metabolism of stem cells in a label-free and non-invasive way measuring metabolic trajectories during differentiation [183, 202, 203, 208–210]. 2P-FLIM of NADH was used to characterize the metabolic state of NPCs during differentiation in live cells, observing that the ratio of free to protein-bound NADH strongly correlates with the differentiation state. Undifferentiated NPCs have a glycolytic phenotype characterized by high free/bound NADH, while the OXPHOS phenotype from differentiated neurons is characterized by low free/bound NADH. Hereby, the metabolic signature of NPCs correlates with their differentiation potential, showing that neuronal progenitors and glial progenitors have a different free/bound NADH ratio in line with evidence that energy metabolism and the redox state are important regulators of NSC [211, 212, 181, 213, 214]. Ultimately, multiparametric metabolic imaging has been recently implemented in an efficient way with multicolor multiphoton simultaneous excitation of NADH ad FAD [215, 216] and it could bring new insights into the understanding of the spatial and temporal metabolic patterns during neural differentiation, as NADH and FAD provide complementary information on different metabolic pathways [209]. Using fluorescently labeled epigenetic remodelers or histone modifications, or even genomic regions through for example dCas9-based techniques [217–220], a combination of FLIM-FRET techniques could be implemented, representing new avenues to uncover the intimate relationship between metabolism and epigenome regulation during differentiation in live cells and tissues.

## Conclusions

In conclusion, the process of neural differentiation is a complex and tightly regulated event that involves wide metabolic and epigenetic transitions. Through the regulation of metabolic pathways, the cell generates energy and biosynthetic precursors necessary for stemness maintenance and determination of cell fate. Additionally, epigenetic modifications, such as DNA methylation, histone modifications, and non-coding RNA, play critical roles in gene expression regulation during neural differentiation.

Recent studies have highlighted the importance of the crosstalk between metabolism and epigenetic during early neural development, which have provided critical insights into the mechanisms that govern neural differentiation. These findings have potential implications for understanding the etiology of neurodevelopmental disorders and for developing new therapies. However, there is still much to be learned about the precise mechanisms that underlie metabolic and epigenetic transitions during neural differentiation. Although the intricate relationship between epigenetics and metabolism has been emerging, there are still many outstanding questions. For example, why are histone post-translational modifications and epigenetic marks at certain genomic loci more susceptible to fluctuations in metabolic cofactor availability than others? Another outstanding question is how metabolic signals shape brain DNA methylation during adulthood and how manipulations of the diet could modulate DNA methylation in the adult brain. Additionally, it is still not fully understood how metabolic pathways and epigenetic modifications interact to regulate NSC fate. Finally, it is important to understand how metabolic transitions translate into changes in redox balance, cell signaling, and epigenetics, thereby regulating stem cell activation and differentiation. For example, in light of current knowledge, it is of interest to define the precise mechanisms through which 1C metabolism modulates DNA methylation and subsequently regulates neuronal fate during development. This represents a pivotal yet challenging area of investigation, necessitating the application of cutting-edge methodologies and integrative approaches to unravel the intricate interplay between these biological processes. Further research is needed to unravel the intricacies of these processes and to determine how they are coordinated and regulated. The future prospects are undoubtedly related to advances in technologies such as simultaneous single-cell transcriptomics and metabolomics in real time which will certainly contribute to our understanding of the molecular mechanisms underlying neural differentiation.

## Data Availability

Not applicable.
